# First preclinical evaluation of [^225^Ac]Ac-DOTA-JR11 and comparison with [^177^Lu]Lu-DOTA-JR11, alpha versus beta radionuclide therapy of NETs

**DOI:** 10.1186/s41181-023-00197-0

**Published:** 2023-06-30

**Authors:** Maryana Handula, Savanne Beekman, Mark Konijnenberg, Debra Stuurman, Corrina de Ridder, Frank Bruchertseifer, Alfred Morgenstern, Antonia Denkova, Erik de Blois, Yann Seimbille

**Affiliations:** 1 Department of Radiology and Nuclear Medicine, Erasmus MC Cancer Institute, Erasmus University Medical Center, 3015 GD Rotterdam, The Netherlands; 2 Department of Experimental Urology, Erasmus University Medical Center, 3015 GD Rotterdam, The Netherlands; 3 European Commission, Join Research Centre, 76344 Karlsruhe, Germany; 4 Applied Radiation and Isotopes, Department of Radiation Science and Technology, Faculty of Applied Sciences, Delft University of Technology, Delft, The Netherlands; 5 Life Sciences Division, TRIUMF, Vancouver, BC V6T 2A3 Canada

**Keywords:** SSTR2, DOTA-JR11, Actinium-225, Lutetium-177, Radionuclide therapy

## Abstract

**Background:**

The [^177^Lu]Lu-DOTA-TATE mediated peptide receptor radionuclide therapy (PRRT) of neuroendocrine tumors (NETs) is sometimes leading to treatment resistance and disease recurrence. An interesting alternative could be the somatostatin antagonist, [^177^Lu]Lu-DOTA-JR11, that demonstrated better biodistribution profile and higher tumor uptake than [^177^Lu]Lu-DOTA-TATE. Furthermore, treatment with alpha emitters showed improvement of the therapeutic index of PRRT due to the high LET offered by the alpha particles compared to beta emitters. Therefore, [^225^Ac]Ac-DOTA-JR11 can be a potential candidate to improve the treatment of NETs (Graphical abstract). DOTA-JR11 was radiolabeled with [^225^Ac]Ac(NO_3_)_3_ and [^177^Lu]LuCl_3_. Stability studies were performed in phosphate buffered saline (PBS) and mouse serum. In vitro competitive binding assay has been carried out in U2OS-SSTR2 + cells for ^nat^La-DOTA-JR11, ^nat^Lu-DOTA-JR11 and DOTA-JR11. Ex vivo biodistribution studies were performed in mice inoculated with H69 cells at 4, 24, 48 and 72 h after injection of [^225^Ac]Ac-DOTA-JR11. A blocking group was included to verify uptake specificity. Dosimetry of selected organs was determined for [^225^Ac]Ac-DOTA-JR11 and [^177^Lu]Lu-DOTA-JR11.

**Results:**

[^225^Ac]Ac-DOTA-JR11 has been successfully prepared and obtained in high radiochemical yield (RCY; 95%) and radiochemical purity (RCP; 94%). [^225^Ac]Ac-DOTA-JR11 showed reasonably good stability in PBS (77% intact radiopeptide at 24 h after incubation) and in mouse serum (~ 81% intact radiopeptide 24 h after incubation). [^177^Lu]Lu-DOTA-JR11 demonstrated excellent stability in both media (> 93%) up to 24 h post incubation. Competitive binding assay revealed that complexation of DOTA-JR11 with ^nat^La and ^nat^Lu did not affect its binding affinity to SSTR2. Similar biodistribution profiles were observed for both radiopeptides, however, higher uptake was noticed in the kidneys, liver and bone for [^225^Ac]Ac-DOTA-JR11 than [^177^Lu]Lu-DOTA-JR11.

**Conclusion:**

[^225^Ac]Ac-DOTA-JR11 showed a higher absorbed dose in the kidneys compared to [^177^Lu]Lu-DOTA-JR11, which may limit further studies with this radiopeptide. However, several strategies can be explored to reduce nephrotoxicity and offer opportunities for future clinical investigations with [^225^Ac]Ac-DOTA-JR11.

**Supplementary Information:**

The online version contains supplementary material available at 10.1186/s41181-023-00197-0.

## Background

Neuroendocrine tumors (NETs) are an indolent and well-differentiated type of neuroendocrine neoplasms (NENs) (Rizen and Phan 2022; Das and Dasari 2021). Most commonly appearing in the gastroenteropancreatic (GEP) system, those malignancies are remaining rare (worldwide, 35 out of 100,000 people are diagnosed yearly) (Öberg and Castellano 2011; Singh et al. 2017). NETs are known to express 5 subtypes of somatostatin receptor (SSTR 1–5) (Mizutani et al. 2012). However, the high expression of SSTR2 by NETs makes it an ideal target for imaging and therapy (Elf et al. 2021; Tafreshi et al. 2021; Rufini et al. 2022; Fonti et al. 2022). SSTR2-mediated peptide receptor radionuclide therapy (PRRT) has been widely employed for the treatment of NETs. Many studies using either somatostatin agonists (e.g., DOTA-TATE and DOTA-TOC) or antagonists (e.g., DOTA-JR11 and DOTA-LM3) were reported (Zhu et al. 2021; Albrecht et al. 2021; Thakur et al. 2021). [^177^Lu]Lu-DOTA-TATE (Lutathera^®^) was recently approved by the Food and Drug Administration (FDA) as the first radiopharmaceutical for the treatment of GEP-NETs. PRRT of NETs using [^177^Lu]Lu-DOTA-TATE showed positive outcomes by increasing the overall survival and improving the quality of life of the patients (Strosberg et al. 2018; Ianniello et al. 2016). However, treatment resistance of NETs indicates that PRRT became less effective due to upregulated DNA damage repair (Katona et al. 2017; Mariniello et al. 2016).

Several studies reported that DOTA-JR11 exhibited lower binding affinity to SSTR2 compared to DOTA-TATE (Hou et al. 2022; Zhu et al. 2020; Fani et al. 2017). However, preclinical and clinical studies revealed a higher tumor uptake with the antagonist, due to its ability to bind to more binding sites on the receptor than the agonist (Fani et al. 2012; Wild et al. 2014). Nevertheless, Reidy-Lagunes et al. reported an increased hematologic toxicity with [^177^Lu]Lu-DOTA-JR11 in comparison to [^177^Lu]Lu-DOTA-TATE (Reidy-Lagunes et al. 2019). Nevertheless, it was found that DOTA-JR11 is a promising SSTR2-antagonist for targeted radionuclide therapy of NETs. Recently, targeted alpha therapy (TAT) demonstrated to be more effective than the standard PRRT, due to the high linear energy transfer (LET) offered by the alpha particles compared to beta emitters (80–100 vs. 0.1–1.0 keV/μm, respectively) (Müller et al. 2017; Navalkissoor and Grossman 2019; Brechbiel 2007). In fact, it was confirmed that alpha particles can induce more DNA damage compared to beta emitters, hence leading to more cell death (Feijtel et al. 2020; Miederer et al. 2008). Furthermore, unlike PRRT where hypoxia can lead to treatment resistance, the use of alpha radiation does not require the presence of oxygen to create effective DNA damage. Thus, several alpha particles-emitters were introduced as good candidates for TAT (e.g., bismuth-213, lead-212). However, actinium-225 has gained a lot of attention due to its long half-life (t_1/2_ = 9.92 days) and interesting decay chain offering 4 alpha particles (Tafreshi et al. 2019; Parker et al. 2018).

Thus, we report herein optimized radiolabeling conditions using various quenchers (e.g., gentisic/ascorbic acids, ethanol and l-melatonin) to reduce or prevent radiolysis. Due to the high LET of alpha particles, different studies reported the instability of biomolecules to alpha radiation. Therefore, we evaluated the stability of [^225^Ac]Ac-DOTA-JR11 towards radiolysis in PBS buffer and enzymatic degradation in mouse serum. Chemical modifications performed at the *N*-terminal of DOTA-JR11 were previously reported to affect the binding affinity of the peptide to SSTR2. Therefore, we evaluated the influence of metal complexation with different nuclides on SSTR2 binding affinity. Furthermore, preclinical evaluation of [^225^Ac]Ac-DOTA-JR11 in tumor bearing mice was carried out. The pharmacokinetic and biodistribution profiles of [^225^Ac]Ac-DOTA-JR11 were compared to the previously published data for [^177^Lu]Lu-DOTA-JR11. Besides, we performed dosimetry studies to compare the therapeutic efficacy of [^225^Ac]Ac-DOTA-JR11 and [^177^Lu]Lu-DOTA-JR11. Murine dosimetry calculations were performed using the OLINDA phantom and MIRD S-values.

## Materials and methods

### General information

The chemicals and solvents were purchased from commercial suppliers and used without further purification. Lanthanum (III) chloride hydrate (99.9%) and lutetium (III) chloride (99.9%) were purchased from Sigma Aldrich (Amsterdam, The Netherlands) and abcr (Karlsruhe, Germany), respectively. Lutetium-177 (non-carrier added, [^177^Lu]LuCl_3_) with a specific activity of 4081 GBq/mg, was purchased from ITM (München, Germany). Actinium-225, with a specific activity of 2150 GBq/mg, was provided by the Joint Research Centre (JRC, Karlsruhe, Germany). [^111^In]InCl_3_ (370.0 MBq/mL in HCl, pH 1.5–1.9) was provided by Curium (Petten, The Netherlands). High-performance liquid chromatography (HPLC) and mass spectrometry (MS) were carried out on a LC/MS 1260 Infinity II system from Agilent (Middelburg, The Netherlands). Analyses were performed on an analytical column (Poroshell 120, EC-C18, 2.7 µm, 3.0 × 100 mm) from Agilent with a gradient elution of acetonitrile (ACN) (5–100% in H_2_O, containing 0.1% formic acid) at a flow rate of 0.5 mL/min over 8 min. Purification of the complexed DOTA-JR11 peptides was carried out on a preparative HPLC 1290 Infinity II system from Agilent using a preparative Agilent 5 Prep C18 column (50 × 21.2 mm, 5 µm). Both compounds were purified using a gradient elution of ACN (10–95% in H_2_O, containing 0.1% formic acid) at a flow rate of 10 mL/min over 10 min. Instant thin-layer chromatography on silica-gel-impregnated glass fiber iTLC-SG sheets (Agilent; Folsom, CA, USA) were eluted with sodium citrate (0.1 M, pH 5). The radioactive samples used to determine the radiochemical yield (RCY), radiochemical purity (RCP) and in vivo studies were counted using a Wizard 2480 gamma counter (Perkin Elmer; Waltham, MA, USA). Quality control of [^225^Ac]Ac-DOTA-JR11 and analysis of its stability were carried out on a HPLC Alliance system from Waters (Etten-Leur, The Netherlands) equipped with a diode array detector, a Canberra Osprey multichannel analyzer (Zelik, Belgium), and an analytical C18 Symmetry^®^ column (Waters; 250.0 × 4.6 mm, 5 µm) eluted with a gradient of methanol (0–100% in H_2_O, containing 0.1% trifluoroacetic acid) at a flowrate of 1 mL/min over 25 min. HPLC fractions (1 fraction/30 s) were collected using an automated fraction collector III from Waters (Etten-Leur, The Netherlands). Determination of the radioactivity in the fractions was performed using a Wizard 2480 gamma counter at least 30 min after collection (Additional file 1: Fig. S2). The detector (thallium activated sodium iodide crystal) was calibrated for francium-221 energy window (186–226 keV) and each fraction was counted for 1 min (Hooijman et al. 2021). Determination of the injected activity of [^225^Ac]Ac-DOTA-JR11 for biodistribution studies was performed using High Purity Germanium (HPGe) detector from Miron Technologies Canberra (Olen, Belgium). Quality control of [^177^Lu]Lu-DOTA-JR11 and analysis of its stability were carried out on an ultra-high performance liquid chromatography (UHPLC) Acquity Arc system from Waters equipped with a diode array detector, a Canberra Osprey multichannel analyzer, and an analytical C18 Gemini^®^ column (250.0 × 4.6 mm, 5 µm) from Phenomenex (Torrance, CA, USA) eluted with a gradient of ACN (5–95% in H_2_O, containing 0.1% trifluoroacetic acid) at a flowrate of 1 mL/min over 30 min.

### Chemistry

#### Complexation of DOTA-JR11 with natural lanthanum and lutetium

DOTA-JR11 was synthesized as previously described (Fani et al. 2011; Cescato et al. 2008). ^nat^La and ^nat^Lu complexes were prepared using an excess (15 equiv.) of ^nat^LaCl_3_ and ^nat^LuCl_3_ in sodium acetate buffer (100 mM, pH 6) and DOTA-JR11 (2.36 µmol). The mixtures were incubated at 45 °C for 1 h. The complexed peptides were separated from the free metal ions by preparative-HPLC purification. ^nat^La-DOTA-JR11 was obtained as a white solid (3.0 mg, 70%). Analytical HPLC retention time of ^nat^La-DOTA-JR11: *t*_R_ = 3.61 min (Additional file 1: Fig. S1A), purity > 97%; ESI-MS: *m*/*z*, calculated: 1825.16, found: 913.50 [M + 2H]^2+^ (Additional file 1: Fig. S1B). ^nat^Lu-DOTA-JR11 was obtained as a white solid (3.0 mg, 68%). Analytical HPLC retention time of ^nat^Lu-DOTA-JR11: *t*_R_ = 3.52 min (Additional file 1: Fig. S1C), purity > 97%; ESI-MS: *m*/*z*, calculated: 1861.22, found: 931.50 [M + 2H]^2+^ (Additional file 1: Fig. S1D).

### Radiochemistry

#### Actinium-225 radiolabeling of DOTA-JR11

[^225^Ac]Ac(NO_3_)_3_ was obtained as a powder, and dissolved in 0.1 M hydrochloride acid (HCl) before use. DOTA-JR11 (10 nmol) was labeled at a molar activity of 50 kBq/nmol in a solution containing gentisic/ascorbic acids (10 µL, 50 mM), sodium acetate (1 µL, 2.5 M, pH 8), DOTA-JR11, ethanol (10 µL), MilliQ water (90.5 µL) supplemented with kolliphor^®^ HS 15 (2.0 mg/mL), and [^225^Ac]Ac(NO_3_)_3_ (2.16 µL, 500 kBq) (condition 1, Table 1). The radiolabeling mixture was incubated at 90 °C for 20 min. Diethylenetriaminepentaacetic acid (DTPA, 5 µL, 4 mM) was added after labeling to complex the free actinium-225. The radiochemical yield (RCY) was determined by instant thin-layer chromatography (iTLC). The iTLC strip was cut into pieces, which were counted in the gamma counter. To determine the radiochemical purity, 100 µL of [^225^Ac]Ac-DOTA-JR11 (10 kBq) diluted in water containing Kolliphor^®^ HS 15 (2.0 mg/mL) were injected into HPLC. Fractions were collected and counted in the gamma counter. The data collected from the iTLC strip or the HPLC fractions were presented as francium-221 counts per centimeter or fraction, respectively.

**Table 1:** Summary of the radiolabeling conditions

	Condition 1	Condition 2	Condition 3
MilliQ water		10 μL	10 μL
MilliQ water containing Kolliphor^®^ HS 15 (2.0 mg/mL)	90.5 μL		
TRIS buffer containing the peptide (0.1 M, pH 9)		75 μL	75 μL
Ascorbate buffer (1.0 M, pH 5.8)		50 μL	50 μL
l-melatonin (0.5 M)			10 μL
Gentisic/ascorbic acids (50 mM)	10 μL		
Sodium acetate (2.5 M, pH 8)	1 μL		
Ethanol	10 μL		
Molar activity	50 kBq/nmol	100 kBq/nmol	100 kBq/nmol
DTPA (4 mM)	5 μL	5 μL	5 μL

[^225^Ac]Ac-DOTA-JR11 was prepared following two other radiolabeling conditions (conditions 2 and 3) at a molar activity of 100 kBq/nmol (Table 1). Radiolabeling of DOTA-JR11 was carried out in TRIS buffer containing the peptide (75 µL, 0.1 M, pH 9), H_2_O (10 µL), ascorbate buffer (50 µL, 1.0 M, pH 5.8) and [^225^Ac]Ac(NO_3_)_3_ dissolved in 0.1 M HCl. Radiolabeling was performed without (condition 2) or with (condition 3) l-melatonin (10 µL, 0.5 M). The labeling mixture (pH ~ 6) was heated at 90 °C for 20 min. The radiochemical yield and purity were determined by iTLC (Additional file 1: Fig. S3) and radio-HPLC, respectively.

#### Lutetium-177 and indium-111 radiolabeling of DOTA-JR11

[^177^Lu]LuCl_3_ was obtained as a 0.05 M hydrochloric acid aqueous solution. A total activity of 50 MBq of either [^177^Lu]LuCl_3_ or [^111^In]InCl_3_ was added to DOTA-JR11 (1 nmol), gentisic/ascorbic acids (10 µL, 50 mM), sodium acetate (1 µL, 2.5 M, pH 8), and kolliphor^®^ HS 15 in H_2_O (2.0 mg/mL, 60.8 µL) (Blois et al. 2014). The mixture was incubated for 20 min at 90 °C and then left to cool down for 5 min. The RCY was determined by iTLC eluted with a solution of sodium citrate buffer (0.1 M, pH 5). DTPA (5 µL, 4 mM) was added to complex free lutetium-177 or indium-111. The RCP of [^177^Lu]Lu-DOTA-JR11 was determined by radio-HPLC (Additional file 1: Fig. S4).

#### Stability studies in PBS and mouse serum of [^225^Ac]Ac-DOTA-JR11 and [^177^Lu]Lu-DOTA-JR11

[^225^Ac]Ac-DOTA-JR11 and [^177^Lu]Lu-DOTA-JR11 (50 kBq and 1.2 MBq, respectively) were incubated in phosphate buffered saline (PBS; 500 and 200 µL, respectively) and mouse serum (Merck; Haarlerbergweg, The Netherlands) (250 and 100 µL, respectively) at 37 °C. The stability of [^225^Ac]Ac-DOTA-JR11 was verified in PBS and mouse serum at 22 h, 24 h and 27 h after incubation at 37 °C for condition 1, 2 and 3, respectively. The stability of [^177^Lu]Lu-DOTA-JR11 was monitored in PBS and mouse serum at 2 and 24 h post incubation. In mouse serum, the proteins were precipitated by adding an aliquot of the radiotracer to an equal volume of acetonitrile. The vial was vortexed vigorously and centrifuged for 20 min at 10,000 rpm. Stability studies were monitored by radio-HPLC (Additional file 1: Figs. S5 and S6).

### In vitro*** evaluation of ***^***nat***^***La-DOTA-JR11 and***^***nat***^***Lu-DOTA-JR11***

#### Cell line and culture

Human osteosarcoma cells (U2OS) transfected with SSTR2 receptor were used for the cell-based competitive binding assays. Cells were cultured in Dulbecco’s modified Eagle’s medium (DMEM) from Gibco (Paisley, UK) supplemented with 2 mM l-glutamine, 10% fetal bovine serum (FBS), 50 units/mL penicillin, and 50 µg/mL streptomycin (Sigma Aldrich; Haarlerbergweg, The Netherlands) and maintained at 37 °C and in a 5% CO_2_ humidified chamber. Passages were performed weekly using trypsin/ethylenediaminetetraacetic acid (trypsin/EDTA) (0.05%/0.02% *w*/*v*).

#### Competition binding assay

Competitive binding experiments against [^111^In]In-DOTA-JR11 were performed with DOTA-JR11, ^nat^La-DOTA-JR11 and ^nat^Lu-DOTA-JR11 in U2OS.SSTR2 cells (Dalm and Jong 2017). Cells were seeded in a 24-well plate 24 h in advance (2 × 10^5^ cells/well). On the day of the experiment, medium was removed, and the cells were washed once with PBS (Gibco). Then, solutions containing unlabeled compound DOTA-JR11, ^nat^La-DOTA-JR11 or ^nat^Lu-DOTA-JR11 in increasing concentrations (10^−12^ to 10^−5^ M) in internalization medium (DMEM media, 20 mM HEPES, 1% BSA, pH 7.4) were added, followed by the addition of [^111^In]In-DOTA-JR11 (10^−9^ M). Experiments were performed in triplicate for each concentration. Cells were incubated at 37 °C for 90 min. After incubation, medium was removed, and cells were washed twice with PBS and lysed with 1.0 M sodium hydroxide (NaOH) for 15 min at rt. The lysate was transferred to counting tubes, and measurement was performed using the gamma counter. IC_50_ values were determined by non-linear regression plot analysis using Graphpad Prism v5 (GraphPad software, San Diego, CA, USA). Data were reported as percentage binding of [^111^In]In-DOTA-JR11.

### Ex vivo* studies*

#### Ex vivo biodistribution of [^225^Ac]Ac-DOTA-JR11

All animal experiments were approved by the animal welfare body of the Erasmus Medical Center, and were performed according to accepted guidelines. BALB/cAnN Rj-Nude mice were inoculated subcutaneously with 5 × 10^6^ SSTR2-positive H69 cells (human small-cell lung carcinoma) in Matrigel (Corning, NY, USA). The tumors were left to grow for approximatively two weeks until reaching an average volume of 300 mm^3^. Then, the animals were intravenously (i.v.) injected through the tail vein with 100 µL of [^225^Ac]Ac-DOTA-JR11 (23.4 ± 1.5 kBq/0.5 nmol) diluted in PBS containing Kolliphor^®^ HS 15 (0.06 mg/ mL) (*n* = 3 mice/group). Ex vivo biodistribution studies were performed at 4, 24, 48 and 72 h post injection (p.i.). The radioactivity uptake of the following organs was determined: blood, tumor, heart, lungs, liver, spleen, stomach, intestines, pancreas, kidneys, muscle, skin and bone. To confirm uptake specificity of [^225^Ac]Ac-DOTA-JR11, a group of mice were co-injected with [^225^Ac]Ac-DOTA-JR11 and a 50-fold excess of unlabeled DOTA-JR11 24 h prior the organs were harvested. The weight of the tissues was measured and the activity present in each organ and tumor was counted in the gamma counter 24 h later. The results were reported as percentage of injected activity per gram of tissue (% IA/g).

#### Ex vivo biodistribution of [^177^Lu]Lu-DOTA-JR11

The ex vivo biodistribution data of [^177^Lu]Lu-DOTA-JR11 were previously published by our group (Koustoulidou et al. 2022). Briefly, BALB/cAnN Rj-Nude mice (4 mice/group) inoculated with SSTR2-positive H69 cells were injected intravenously, through the tail vein, with 100 µL of [^177^Lu]Lu-DOTA-JR11 (5 MBq/0.5 nmol). Ex vivo biodistribution studies were performed at 4, 24, 48 and 72 h p.i. Uptake specificity of [^177^Lu]Lu-DOTA-JR11 was confirmed by co-injection of the radiopeptide with a 50-fold excess of unlabeled DOTA-JR11. The harvested organs of interest were counted in a gamma counter and data were reported as % IA/g (Additional file 1: Table S2).

#### Digital autoradiography of tumor and kidney slices

Tumor and kidneys were harvested from one mouse 24 h after administration of 23.4 ± 1.5 kBq/0.5 nmol of [^225^Ac]Ac-DOTA-JR11. The tissues were directly stored in KP-CryoCompound a frozen tissues medium from Klinipath (Olen, Belgium). After being embedded in optimal cutting temperature, the tissues were cut into 10 µm sections using Cryostar NX70 from Thermo Fisher scientific (Eindhoven, The Netherlands). Digital autoradiography images were obtained using BeaQuant from Ai4r (Nantes, France). Image acquisition and image analysis were performed using Beavacq and Beamage, respectively, provided by Ai4r (Nantes, France).

### Statistical analysis

All statistical analysis and nonlinear regression were performed using GraphPad Prism 9.3.1 (San Diego, CA, USA). Outliners were evaluated with the Grubbs’ test and removed from the data set. Significant differences were determined using an unpaired *t*-test. Data were reported as mean ± SEM (standard error of mean) for three independent replicates.

### Dosimetry studies

The absorbed dose was calculated according to the MIRD-scheme [MIRD pamphlet 21]. Single-exponential curves were fitted to the biodistribution data and decision on inclusion of a residual activity was based on the Aikake Information Criterion using Graphpad Prism. The resulting time-activity curves (TAC) were folded with the actinium-225 decay function and integrated over time to determine the time-integrated activity concentration coefficient *[ã(r*_*s*_*)]* for each source organ *r*_*s.*_ The daughters of actinium-225 were assumed to be in equilibrium with actinium-225 activity and the same time-integrated activity concentrations *[ã(r*_*s*_*)]* were applied for each daughter except polonium-213 and thallium-209, which were corrected for their decay branching ratio of 0.9786 and 0.0214, respectively. Absorbed dose coefficients *d(r*_*t*_*)* to target organ r_t_ were calculated by the MIRD equation:

$$d\left( {r_{`t} } \right) = \mathop \sum \limits_{{r_{s} }} \left[ {\tilde{a}\left( {r_{s} } \right)} \right]m\left( {r_{s} } \right) \times S_{RBE} \left( {r_{t} \leftarrow r_{s} } \right)$$

using the radiation RBE weighted S-values for actinium-225 and its progeny and the source organ masses *m(r*_*s*_*)* for the 25 g mouse phantom from the Olinda dosimetry software (Version 2.1, Hermes software) (Andersson et al. 2017). The default RBE of 5 was used for α-radiation and RBE = 1 for all other radiations. The absorbed dose to the tumor was determined by taking the S-value for a 0.15 cm^3^ water sphere from IDAC-dose (version 2.1), again with RBE = 5 for α-radiation and RBE = 1 for all other. The cross-doses from other source organs to the tumor were calculated by adding the cross doses in the testes to the self-dose of the tumor, as both testes and tumor are superficial on the mouse body.

The dosimetry calculations for [^177^Lu]Lu-DOTA-JR11 were performed in the same manner, except that for lutetium-177, RBE = 1. The biodistribution data of [^177^Lu]Lu-DOTA-JR11 was also fitted with single-exponential curves and the resulting time-integrated activity concentration coefficients used to calculate the absorbed doses inside 25 g Olinda mouse phantom. Tumor dosimetry was based on the spheres S-values for actinium-225 with daughters taken from the IDAC code (Andersson et al. 2017).

## Results

### ***Synthesis of ***^***nat***^***La-DOTA-JR11 and***^***nat***^***Lu-DOTA-JR11***

Complexation of DOTA-JR11 with ^nat^LaCl_3_ and ^nat^LuCl_3_ was successfully performed using sodium acetate buffer. The complexes, ^nat^La-DOTA-JR11 and ^nat^Lu-DOTA-JR11 were obtained in high chemical purity (> 97%) and yields of 70 and 68%, respectively.

### ***Radiolabeling and stability studies of [***^***225***^***Ac]Ac-DOTA-JR11 and [***^***177***^***Lu]Lu-DOTA-JR11***

Labeling of DOTA-JR11 was successfully performed with [^225^Ac]Ac(NO_3_)_3_ and [^177^Lu]LuCl_3_ using a mixture of gentisic/ascorbic acids and ethanol as radiolysis quenchers (Blois et al. 2014). Kolliphor^®^ HS 15 was employed during the labeling to reduce the stickiness encountered with the radiolabeled peptides (Koustoulidou et al. 2022). The use of l-melatonin in condition 3 did not only improve the RCP of [^225^Ac]Ac-DOTA-JR11 compared to condition 2, but also improved its stability towards radiolysis (81.0% vs. 56.6%, respectively) (Blois et al. 2013). [^225^Ac]Ac-DOTA-JR11 from condition 1 and [^177^Lu]Lu-DOTA-JR11 were both obtained with a RCP of > 92% (Tables 2 and 3). [^177^Lu]Lu-DOTA-JR11 showed a better stability in PBS and mouse serum compared to the ^225^Ac-labeled analog. Considering our stability data, DOTA-JR11 was radiolabeled with [^225^Ac]Ac(NO_3_)_3_ following condition 1 for the in vivo studies.

**Table 2:** RCYs, RCPs and stability studies in PBS and mouse serum of [^225^Ac]Ac-DOTA-JR11 prepared following different radiochemical conditions: 1, 2 (without l-melatonin) and 3 (with l-melatonin)

	RCY (%)*	RCP (%)*	PBS (%)^†^	Mouse serum (%)^†^
Condition 1	95.1 ± 0.3	92.5 ± 3.8	76.9^a^	80.9^a^
Condition 2	86.9 ± 13.2	76.5 ± 14.7	56.6^b^	81.4^b^
Condition 3	98.4 ± 0.5	83.3 ± 7.5	81.0^c^	81.0^c^

*RCYs and RCPs are presented as percentage of radiolabeled peptide (n = 4, 3 and 4 for conditions 1, 2 and 3 respectively)

^†^Results are expressed as percentage (%) of intact radiolabeled peptide after incubation at 37 °C (n = 1)

^a^Stability studies performed at 22 h

^b^Stability studies performed at 24 h

^c^Stability studies performed at 27 h

**Table 3:** RCY, RCP and stability studies of [^177^Lu]Lu-DOTA-JR11 in PBS and mouse serum

	RCY (%)	RCP (%)	PBS (%)*		Mouse serum (%)*	
			2 h	24 h	2 h	24 h
[^177^Lu]Lu-DOTA-JR11	99.0	96.6	96.5	96.8	94.5	93.7

*Results are expressed as percentage (%) of intact radiolabeled peptide after incubation at 37 °C (n = 1)

### Competition binding assay

**Fig. 1:**
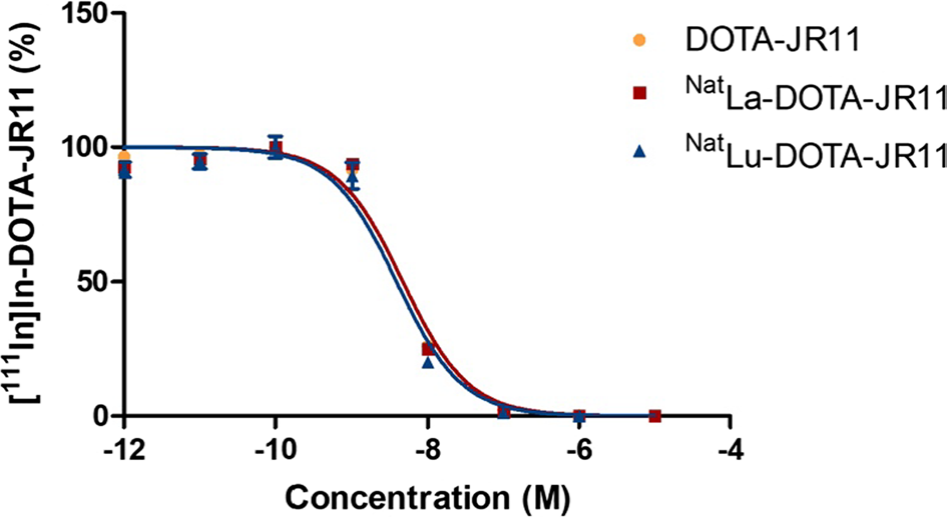
IC_50_ curves of ^nat^La-DOTA-JR11, ^nat^Lu-DOTA-JR11 and DOTA-JR11

The competitive binding assay was carried out in U2OS-SSTR2 + cells and [^111^In]In-DOTA-JR11 was used as radioligand. The obtained IC_50_ curves are reported in Fig. 1. DOTA-JR11, ^nat^La-DOTA-JR11 and ^nat^Lu-DOTA-JR11 exhibited IC_50_ values of 4.69 ± 0.03, 4.71 ± 0.04 and 3.88 ± 0.05 nM, respectively.

### Ex vivo*** biodistribution of [***^***225***^***Ac]Ac-DOTA-JR11***

**Fig. 2:**
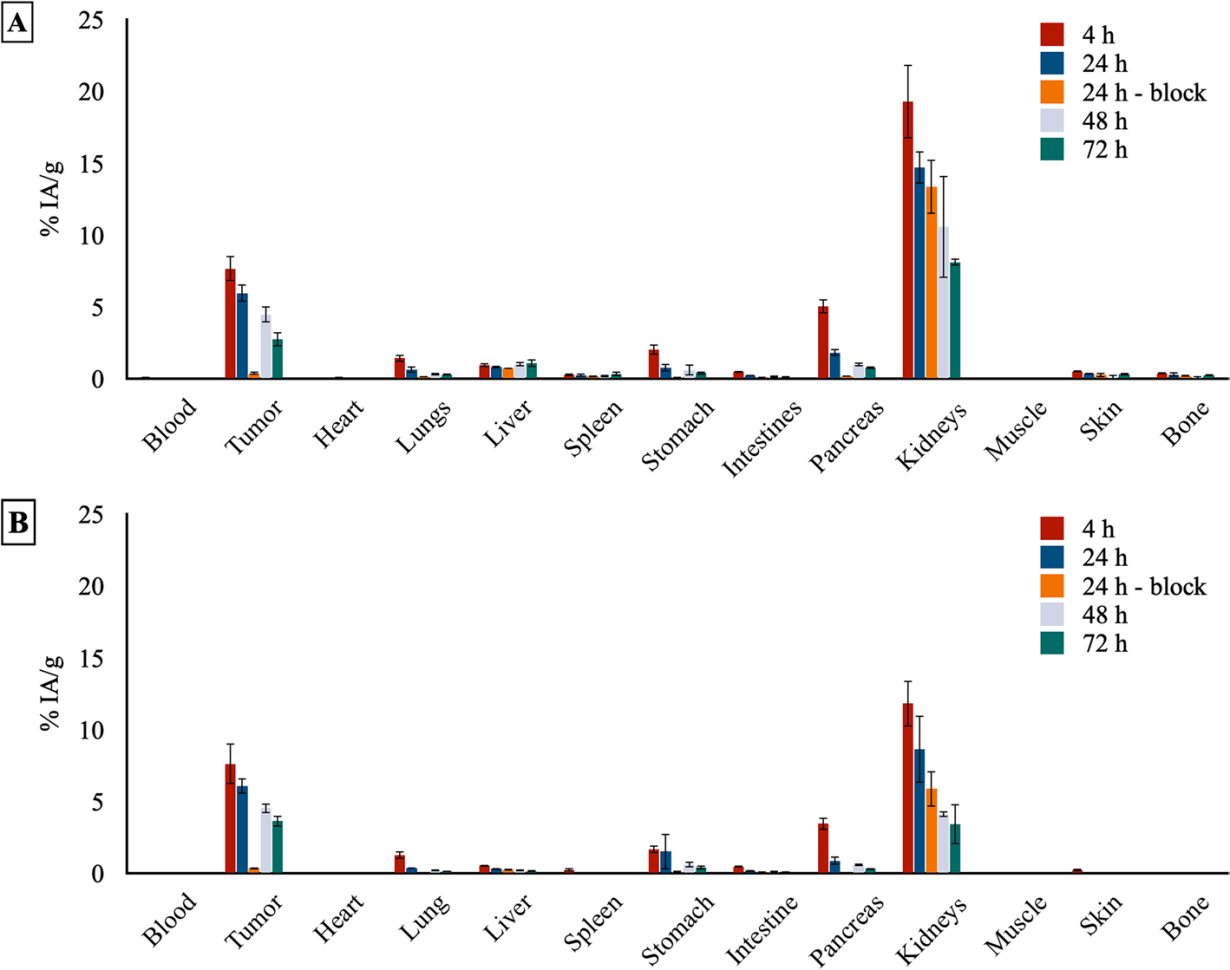
Ex-vivo biodistribution of **A** [^225^Ac]Ac-DOTA-JR11 and **B** [^177^Lu]Lu-DOTA-JR11 at 4, 24, 48 and 72 h post-injection (n = 3 mice/group). Data are presented as the percentage of injected activity per gram of tissue (% IA/g)

Each mouse was injected with 23.4 ± 1.5 kBq/0.5 nmol of radiolabeled peptide and ex vivo biodistribution studies were performed at 4, 24, 48 and 72 h p.i. of [^225^Ac]Ac-DOTA-JR11. A high tumor uptake (7.7 ± 0.9% IA/g) was observed at 4 h p.i. (Fig. 2 and Additional file 1: Table S1), but the radiopeptide was slowly cleared from the tumor to reach an uptake of 2.8 ± 0.5% IA/g at 72 h p.i.. The blocking group confirmed the specific uptake of [^225^Ac]Ac-DOTA-JR11 at 24 h p.i. (0.4 ± 0.1% IA/g compared to 6.0 ± 0.6% IA/g for the non-block group). Furthermore, we noticed a high kidney uptake of [^225^Ac]Ac-DOTA-JR11 at 4 h p.i. (19.3 ± 2.6% IA/g), which decreased overtime (8.1 ± 0.3% IA/g at 72 h p.i.). The clearance of [^225^Ac]Ac-DOTA-JR11 from the tumor and the elimination from the kidneys resulted in low and steady tumor-to-kidney ratio (0.4 and 0.3 at 4 and 72 h p.i., respectively). Further statistical analysis and comparison between [^225^Ac]Ac-DOTA-JR11 and [^177^Lu]Lu-DOTA-JR11 are provided in the supplementary information (Additional file 1: Fig. S7).

### Digital autoradiography

**Fig. 3:**
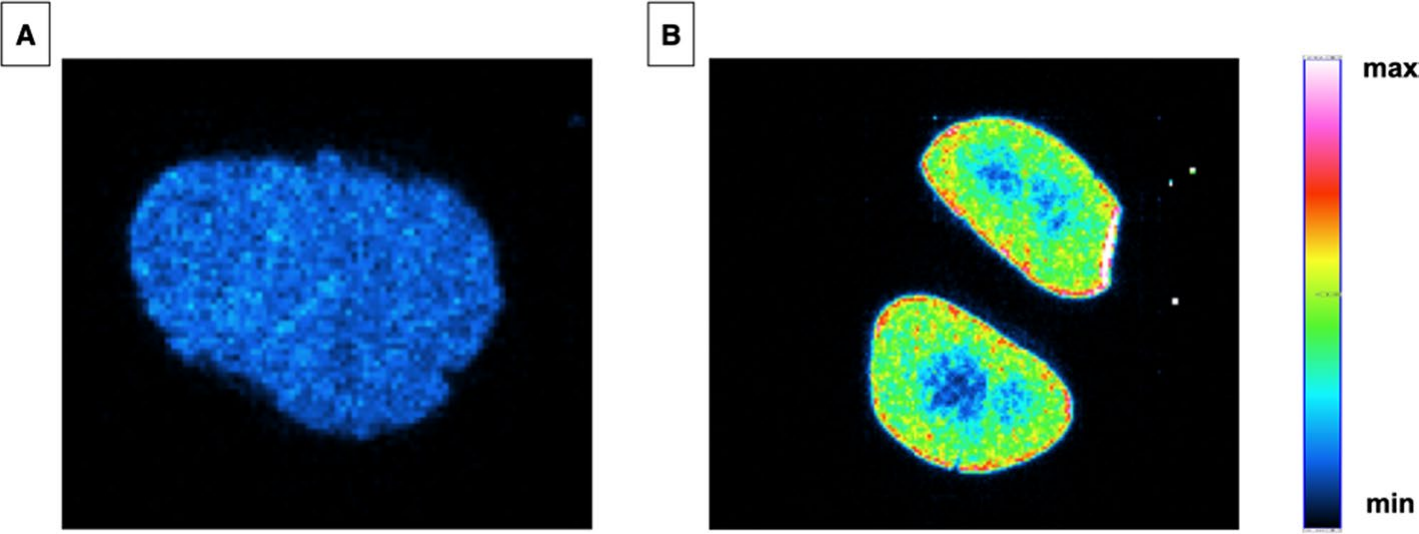
Autoradiography acquisition of **A** a tumor slice and **B** left and right kidneys slice. Tissues were harvested from a mouse 24 h after injection of 23.4 ± 1.5 kBq/0.5 nmol of [^225^Ac]Ac-DOTA-JR11

Tumor and kidneys of one mouse were excised 24 h after administration of [^225^Ac]Ac-DOTA-JR11, sliced and imaged by autoradiography. The images revealed a homogeneous tumor uptake (Fig. 3A). However, heterogenous uptake of [^225^Ac]Ac-DOTA-JR11 was observed in the kidneys (Fig. 3B). In fact, higher uptake was found in the renal cortex compared to the medulla. This finding suggests that the renal distribution of [^225^Ac]Ac-DOTA-JR11 is similar to previously reported somatostatin analogs (Melis et al. 2005).

### Dosimetry studies

**Fig. 4:**
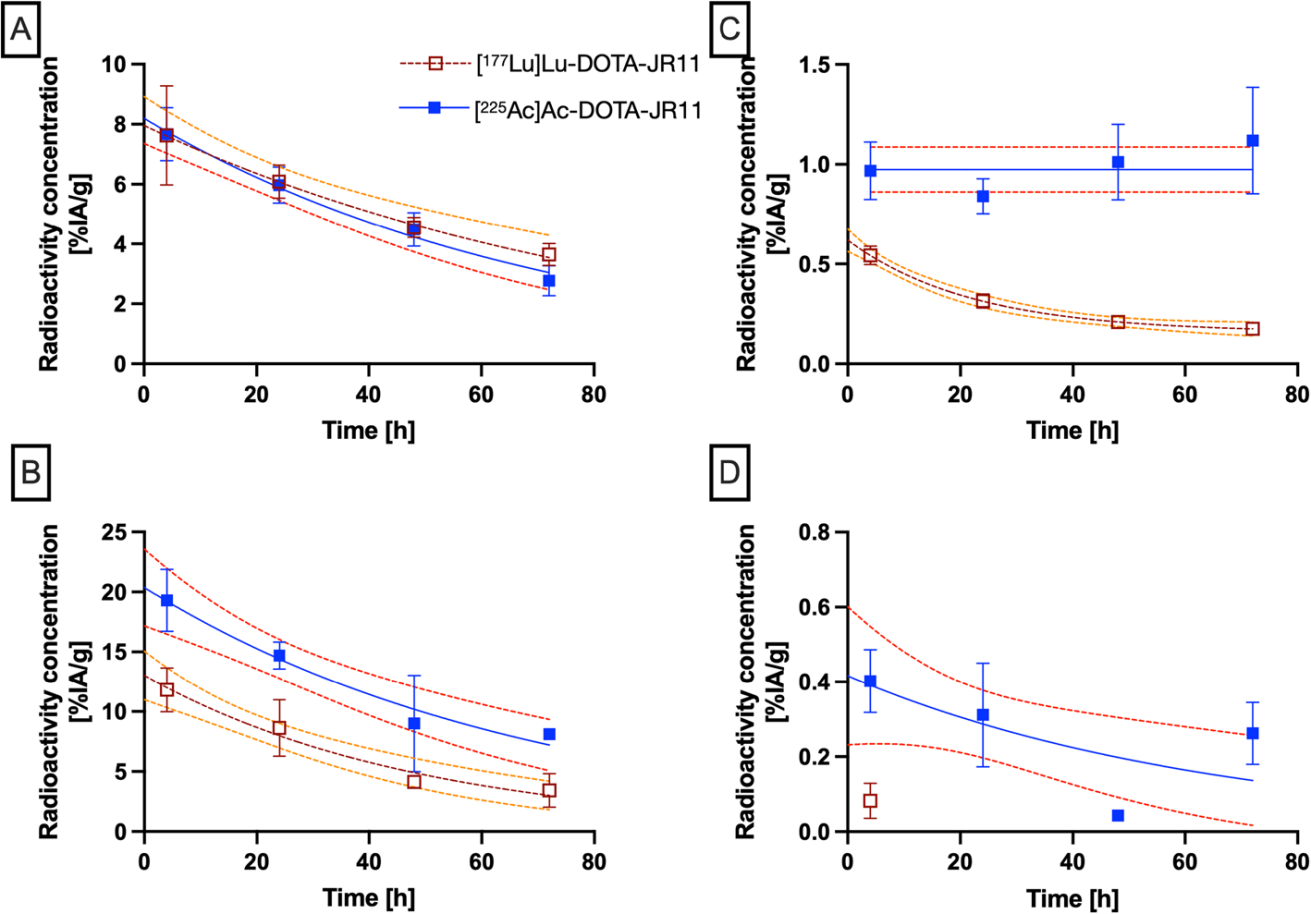
Comparison of **A** tumor, **B** kidneys, **C** liver and **D** bone TAC of [^225^Ac]Ac-DOTA-JR11 (blue curves) and [^177^Lu]Lu-DOTA-JR11 (brown curves). Single-exponential curve fits are shown with 95% confidence intervals

The TACs were fitted with single-exponential functions to the biodistribution data with R^2^ > 0.8, except for liver and bone. The uptake in the liver was considered to be trapped and the TAC was modelled by a horizontal line. The clearance from the tumor proceeded with biological half-lives of 50 h (95% CI 40–65 h) and 62 h (45–93 h) for actinium-225 and lutetium-177, respectively (Fig. 4A). Kidney clearance proceeded with a biological half-life of 48 h (34–75 h) for [^225^Ac]Ac-DOTA-JR11, which is 40% (but not significantly) longer than the 34 h (25–49 h) for [^177^Lu]Lu-DOTA-JR11 (Fig. 4B). Absorbed doses for the organs of interest are presented in Table 4.

**Table 4:** Absorbed dose per administered activity for [^225^Ac]Ac-DOTA-JR11 and [^177^Lu]Lu-DOTA-JR11

Target organ (r_t_)	d(r_t_) [mGy/kBq [^225^Ac]Ac-DOTA-JR11]	d(r_t_) [mGy/MBq [^177^Lu]Lu-DOTA-JR11]
Intestines	40.3	23.4
Stomach Wall	137.2	48.8
Heart	11.2	1.9
Kidneys	952.6	406.9
Liver	271.4	38.5
Lungs	37.2	37.7
Pancreas	284.4	120.8
Skeleton	0.1	2.8
Spleen	76.4	8.4
Total Body	44.0	12.6
Tumor (150 mg)	328.5	464.4
T/K ratio^a^	0.35	1.14

The alpha-contribution to the absorbed dose by [^225^Ac]Ac-DOTA-JR11 were weighted with an RBE = 5. The last row indicates the tumor/kidney absorbed dose ratio

^a^Tumor-to-kidney ratio

Time-activity curves for the organs of interest are presented for [^225^Ac]Ac-DOTA-JR11 and [^177^Lu]Lu-DOTA-JR11 in Additional file 1: Figures S8 and S9 respectively.

## Discussion

Our study aimed to explore the efficacy of TAT in treating NETs using the ^225^Ac-labeled somatostatin antagonist DOTA-JR11. The current gold standard method for PRRT using [^177^Lu]Lu-DOTA-TATE is insufficient due to resistance and recurring disease, and therefore requires improvement (Katona et al. 2017; Mariniello et al. 2016). Alpha particles have a greater ability to induce DNA damage and result in higher cell death compared to beta emitters, making TAT a promising alternative. However, DOTA-JR11 is known to be very sensitive to N-terminus modifications (Fani et al. 2012). Therefore, we first investigated the effect of the complexation with different nuclides on the affinity of the peptide to SSTR2. ^nat^LaCl_3_ was considered as a surrogate for actinium-225 to complex DOTA-JR11 due to identical chemical properties to lanthanide and more specifically La^3+^ (Thiele and Wilson 2018). Competitive binding assay revealed that the complexes, ^nat^La-DOTA-JR11 and ^nat^Lu-DOTA-JR11, exhibited similar binding affinity for SSTR2 compared to the parent peptide DOTA-JR11. These data suggest that complexation of DOTA-JR11 with La^3+^ and Lu^3+^ does not affect the binding affinity of the peptide to SSTR2, which is in agreement with findings previously reported by Fani et al. (Fani et al. 2012; Elkins 2015). Radiolabeling with [^225^Ac]Ac(NO_3_)_3_ is often challenging due to the important degradation of the radiolabeled product caused by the alpha particles. However, we established optimized radiolabeling conditions to obtain [^225^Ac]Ac-DOTA-JR11 in high RCP (condition 1). Lower radiochemical purity and more degradation were observed using condition 2. The use of l-melatonin, as radiolysis quencher (condition 3), was an interesting option to stabilize [^225^Ac]Ac-DOTA-JR11. However, the concentration used during radiolabeling would cause adverse effects during the in vivo preclinical evaluation (Kennaway et al. 2002; Kennaway 2019). Stability studies in PBS and mouse serum revealed that [^225^Ac]Ac-DOTA-JR11 was more sensitive to degradation compared to [^177^Lu]Lu-DOTA-JR11. This is probably due to the higher sensitivity of biomolecules to alpha radiation than beta particles (Pouget and Constanzo 2021; Desouky et al. 2015).

Ex vivo analyses showed that the biodistribution profile of [^225^Ac]Ac-DOTA-JR11 is similar to the biodistribution we previously reported for [^177^Lu]Lu-DOTA-JR11 (Koustoulidou et al. 2022). No statistical difference of tumor uptake could be found between both radiolabeled DOTA-JR11, showing that complexation with lutetium-177 or actinium-225 did not affect tumor uptake in vivo. [^225^Ac]Ac-DOTA-JR11 was completely cleared from the blood circulation 4 h after injection. Uptake in stomach and pancreas was observed, which can be explained by the natural expression of SSTR2 in these organs (Kailey et al. 2012; Watanabe et al. 2022). The specificity of [^225^Ac]Ac-DOTA-JR11 towards SSTR2 was confirmed using the blocking group performed at 24 h after injection, this finding follows the data reported for [^177^Lu]Lu-DOTA-JR11 (Koustoulidou et al. 2022). However, significantly higher kidneys, liver and bone uptake were found for [^225^Ac]Ac-DOTA-JR11 than [^177^Lu]Lu-DOTA-JR11, it might be due to the slight instability of the ^225^Ac-labeled peptide towards radiolysis compared to [^177^Lu]Lu-DOTA-JR11. In fact, it was reported that free actinium-225 or complexed actinium-225 with aminopolycarboxylate chelators distributes mainly to the liver, femur and kidneys (Davis et al. 1999; Yoshimoto et al. 2021). In previous research studies reported by Schwartz and coworkers, it was demonstrated that bismuth-213 generated from the decay of actinium-225 in vivo can accumulate in the kidneys (Schwartz et al. 2011). However, in our current research, the measurement of the organs resulted from the ex vivo biodistribution studies were based on the detection of francium-221 (mother radionuclide of bismuth-213), therefore, the higher kidneys uptake cannot result from an extended retention of bismuth-213.

Considering the tumor/kidney ratio of 0.35 for [^225^Ac]Ac-DOTA-JR11, this seems to prohibit its use as therapeutic agent. However, the absorbed doses were all calculated based on homogeneous uptake in the organs and this can lead to an overestimation of the actual absorbed dose to the glomeruli. In a mouse phantom it was calculated that with actinium-225 located in the proximal tubule the dose to the glomeruli was 57% lower than the mean dose to the kidneys assuming homogenous distribution (Hobbs et al. 2012). In the larger human kidneys this effect is more pronounced and can lead to 81% reduction in absorbed dose to the nephron/glomeruli. Comparable differences were observed between heterogeneous and homogeneous [^177^Lu]Lu-DOTA-TATE absorbed dose distributions (Konijnenberg et al. 2007).

Increased uptake in the femur might lead to higher absorbed doses to the bone marrow, although this is not apparent in the present study. Larger animal models would be needed to determine the bone uptake in further detail, as for instance differentiation between bone and bone marrow. Previous pre-clinical investigations on [^177^Lu]Lu-DOTA-JR11 did not lead to large concerns on its potential hematologic toxicity, whereas the phase 1 clinical trial with [^177^Lu]Lu-DOTA-JR11 was stopped because of severe grade 4 hematologic toxicity. When the skeletal uptake is in the bone structure and not in the bone marrow, the alpha-particles from actinium-225 might be creating less hematopoietic damage than lutetium-177, due to its shorter range. Furthermore, the dosimetry calculations showed that the liver and the pancreas had a 7- and twofold higher absorbed dose for [^225^Ac]Ac-DOTA-JR11 compared to [^177^Lu]Lu-DOTA-JR11, respectively. Based on the results obtained in the current study and the dosimetry calculations, there is a clear need for a better prediction model of bone marrow toxicity in the clinical as in the pre-clinical setting.

The use of [^225^Ac]Ac-DOTA-JR11 in clinical trials may lead to radiotoxicity to non-targeted organs, more specifically to the kidneys. However, several studies showed that kidneys uptake can be reduced using multiple strategies, such as the administration of amino acid cocktails and gelofusine (Eerd et al. 2006; Geenen et al. 2021). Furthermore, introduction of a linker cleaved by the renal brush border enzymes and the pretargeting approach proved their potential in limiting the nephrotoxicity (Arano 2021; Chigoho et al. 2021). Those strategies might reduce the kidneys uptake up to 45–50% offering an opportunity to consider further studies with [^225^Ac]Ac-DOTA-JR11 for TAT of NETs.

## Conclusion

In the current study, we have reported a successful and optimized radiolabeling of DOTA-JR11 with [^225^Ac]Ac(NO_3_)_3_ for TAT of NETs. The radiolabeled peptide was obtained in high RCY and RCP. [^177^Lu]Lu-DOTA-JR11 showed better stability in PBS and mouse serum compared to [^225^Ac]Ac-DOTA-JR11. Complexation of DOTA-JR11 with either ^nat^La or ^nat^Lu did not affect binding affinity to SSTR2. Both radiolabeled peptides showed similar biodistribution profile in vivo. However, due to potential radiotoxicity of the alpha particles in non-targeted organs (e.g., kidneys and bone marrow), further optimization of the pharmacokinetics of [^225^Ac]Ac-DOTA-JR11 are needed for safe and efficient TAT of NETs.

## Supplementary Information

**Additional file 1**. The following supporting information can be found, **Figure S1:** HPLC chromatograms of the complexation of DOTA-JR11 with natural lanthanum and lutetium; **Figure S2:** Percentage ingrowth of francium-221 based on the decay of actinium-225; **Figure S3:** iTLC chromatograms of DOTA-JR11 radio labeled with [225Ac]Ac3 according to conditions 1, 2 and 3; **Figure S4:** radio-HPLC chromatogram of [177Lu]Lu-DOTA-JR11 and UV chromatogram of natLu-DOTA-JR11; **Figure S5:** stability studies of [225Ac]Ac-DOTA-JR11 in PBS and mouse serum; **Figure S6:** Stability studies of [177Lu]Lu-DOTA-JR11 in PBS and mouse serum; **Table S1:** ex vivo biodistribution data of [225Ac]Ac-DOTA-JR11; **Figure S7:** comparison of the tumor, kidneys, liver and bone uptakes of [225Ac]Ac-DOTA-JR11 and [177Lu]Lu-DOTA-JR11; **Table S2:** ex vivo biodistribution data of [177Lu]Lu-DOTA-JR11; Figure S8: time activity curves of the tumor and organs of interest after administration of [225Ac]Ac-DOTA-JR11; **Figure S9:** time activity curves of the tumor and organs of interest after administration of [177Lu]Lu-DOTA-JR11.

## Data Availability

All data generated and analyzed during this study are included in this published article. Supporting information is provided containing additional data. Additional information is available from the corresponding author upon reasonable request.
