# Supplementary material for: First preclinical evaluation of [225Ac]Ac-DOTA-JR11 and comparison with [177Lu]Lu-DOTA-JR11, alpha versus beta radionuclide therapy of NETs
Source: EJNMMI Radiopharm Chem. 2023 Jun 30;8:13. doi: 10.1186/s41181-023-00197-0 (PMC10313624; doi:10.1186/s41181-023-00197-0)
Supplement: Supplementary file 1 — Additional file 1. The following supporting information can be found, Figure S1: HPLC chromatograms of the complexation of DOTA-JR11 with natural lanthanum and lutetium; Figure S2: Percentage ingrowth of francium-221 based on the decay of actinium-225; Figure S3: iTLC chromatograms of DOTA-JR11 radio labeled with [225Ac]Ac3 according to conditions 1, 2 and 3; Figure S4: radio-HPLC chromatogram of [177Lu]Lu-DOTA-JR11 and UV chromatogram of natLu-DOTA-JR11; Figure S5: stability studies of [225Ac]Ac-DOTA-JR11 in PBS and mouse serum; Figure S6: Stability studies of [177Lu]Lu-DOTA-JR11 in PBS and mouse serum; Table S1: ex vivo biodistribution data of [225Ac]Ac-DOTA-JR11; Figure S7: comparison of the tumor, kidneys, liver and bone uptakes of [225Ac]Ac-DOTA-JR11 and [177Lu]Lu-DOTA-JR11; Table S2: ex vivo biodistribution data of [177Lu]Lu-DOTA-JR11; Figure S8: time activity curves of the tumor and organs of interest after administration of [225Ac]Ac-DOTA-JR11; Figure S9: time activity curves of the tumor and organs of interest after administration of [177Lu]Lu-DOTA-JR11. [file 41181_2023_197_MOESM1_ESM.docx]

**Supplemental Information**

**First preclinical evaluation of [^225^Ac]Ac-DOTA-JR11 and comparison with [^177^Lu]Lu-DOTA-JR11, alpha versus beta radionuclide therapy of NETs**

Maryana Handula^1^. Savanne Beekman^1^. Mark Konijnenberg^1^. Debra Stuurman^1,2^. Corrina de Ridder^1,2^. Frank Bruchertseifer^3^. Alfred Morgenstern^3^. Antonia Denkova^4^. Erik de Blois^1^. Yann Seimbille^1,5,*^.

^1^ Department of Radiology and Nuclear Medicine, Erasmus MC Cancer Institute, Erasmus University Medical Center, 3015 GD Rotterdam, The Netherlands

^2^ Department of Experimental Urology, Erasmus University Medical Center, 3015 GD Rotterdam, The Netherlands

^3^ European Commission, Join Research Centre, 76344 Karlsruhe, Germany

^4^ Applied Radiation and Isotopes, Department of Radiation Science and Technology, Faculty of Applied Sciences, Delft University of Technology, Delft, the Netherlands

^5^ Life Sciences Division, TRIUMF, Vancouver, BC V6T 2A3, Canada

^*^ Correspondence: y.seimbille@erasmusmc.nl

Table of Contents

[1. Chemistry 3](#_Toc135168297)

[1.1. Complexation of DOTA-JR11 with lanthanum-139 and lutetium-175 3](#_Toc135168298)

[2. Radiochemistry 3](#_Toc135168299)

[2.1. Actinium-225 radiolabeling of DOTA-JR11 4](#_Toc135168300)

[2.2. Lutetium-177 radiolabeling of DOTA-JR11 4](#_Toc135168301)

[2.3. Stability studies in PBS and mouse serum of [^225^Ac]Ac-DOTA-JR11and [^177^Lu]Lu-DOTA-JR11 5](#_Toc135168302)

[3. Ex vivo studies 6](#_Toc135168303)

[3.1. Ex vivo biodistribution of [^225^Ac]Ac-DOTA-JR11 6](#_Toc135168304)

[3.2. Ex vivo biodistribution of [^177^Lu]Lu-DOTA-JR11 7](#_Toc135168305)

[4. Dosimetry studies 8](#_Toc135168306)

# Chemistry

## Complexation of DOTA-JR11 with natural lanthanum and lutetium


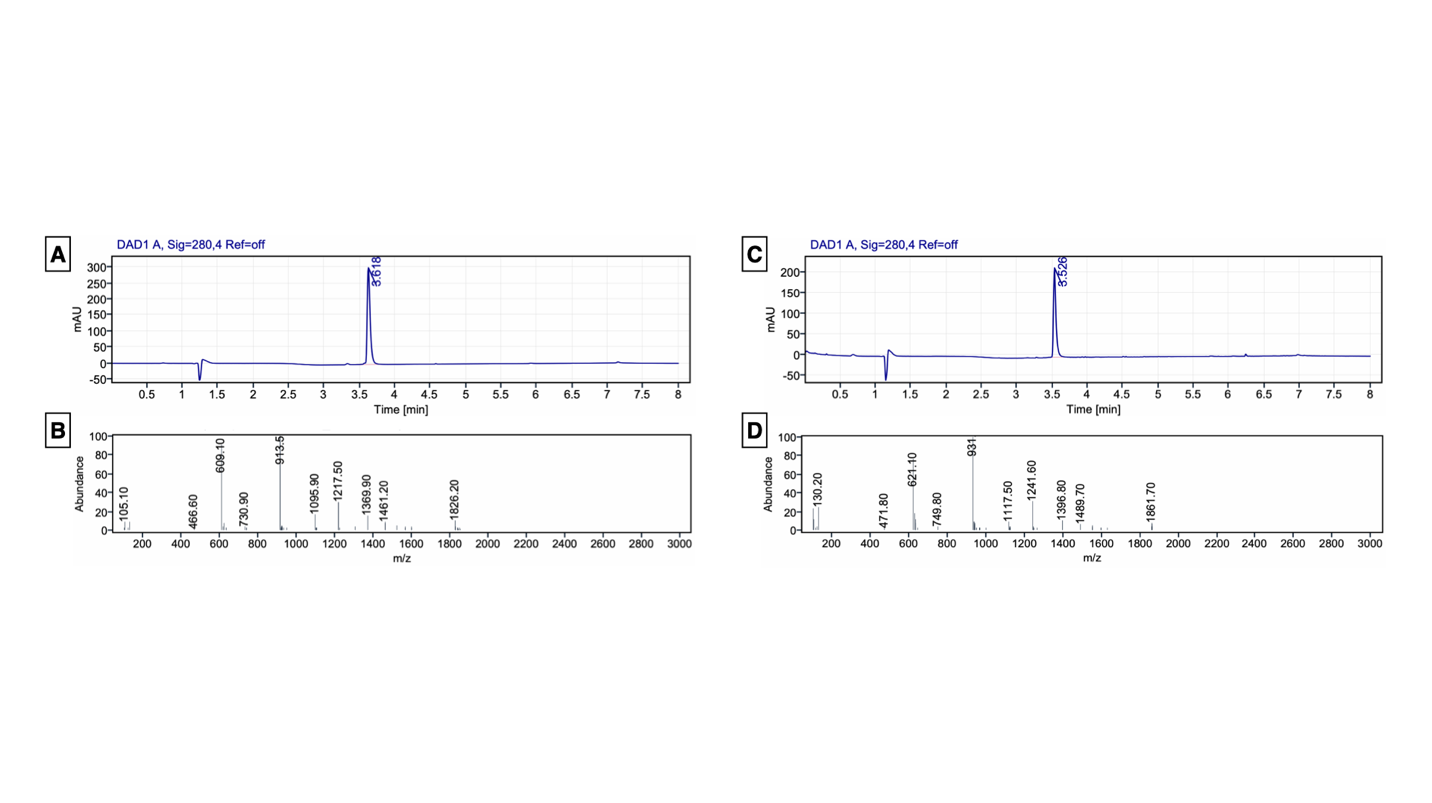


Figure S1: A) and C) HPLC chromatograms and B) and D) mass spectra of ^nat^La-DOTA-JR11 and ^nat^Lu-DOTA-JR11 respectively.

# Radiochemistry

The quality control of all samples containing [^225^Ac]Ac-DOTA-JR11 was based on the measurement of francium-221 in the gamma counter, due to its gamma emission at 218 KeV. Francium-221 is the first daughter radionuclide of actinium-225. Samples were counted at least 30 minutes after collection to ensure that an equilibrium between actinium-225 and francium-221 was achieved (Fig. S2).


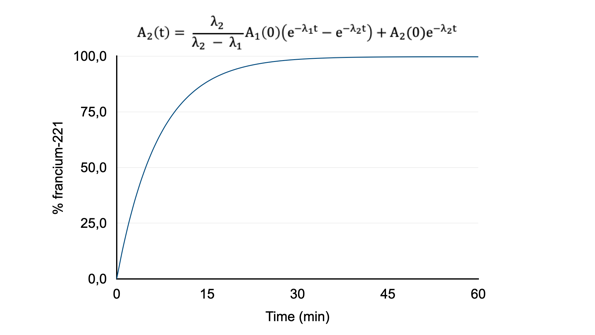


Figure S2:Percentage ingrowth of francium-221 based on the decay of actinium-225 overtime. The curve was calculated based on the mathematical equation shown above.

## Actinium-225 radiolabeling of DOTA-JR11


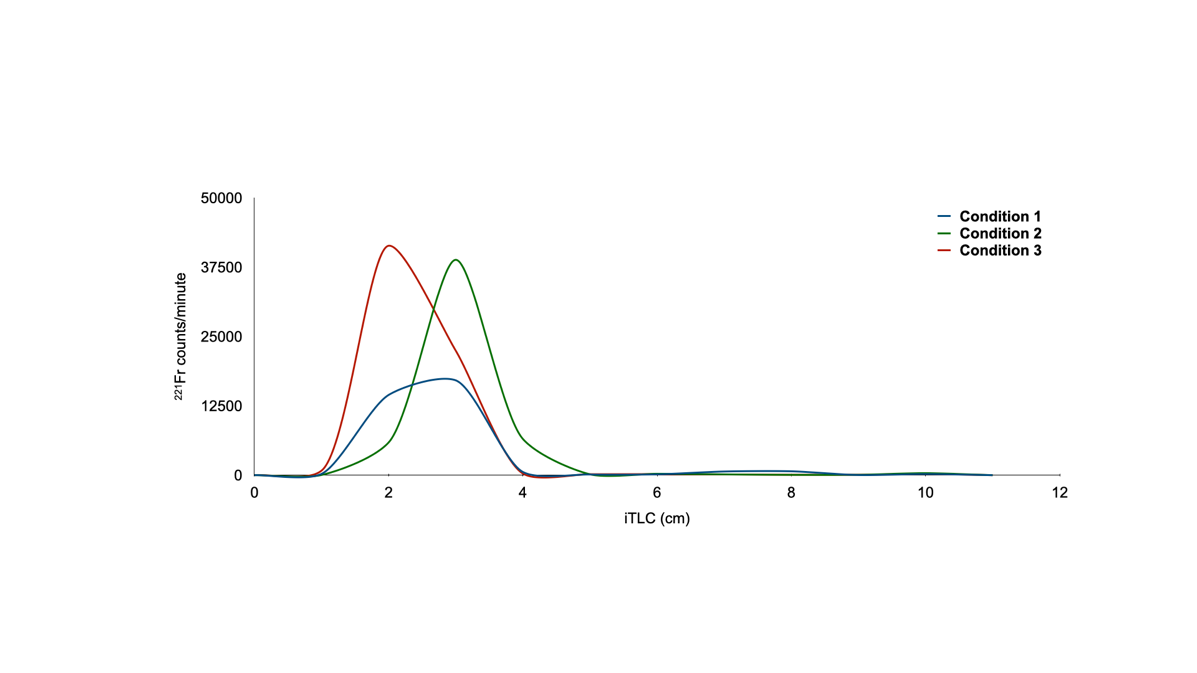


Figure S3: iTLC chromatograms of DOTA-JR11 radiolabeled with [^225^Ac]Ac(NO_3_)_3_ following conditions 1, 2 and 3.

## Lutetium-177 radiolabeling of DOTA-JR11


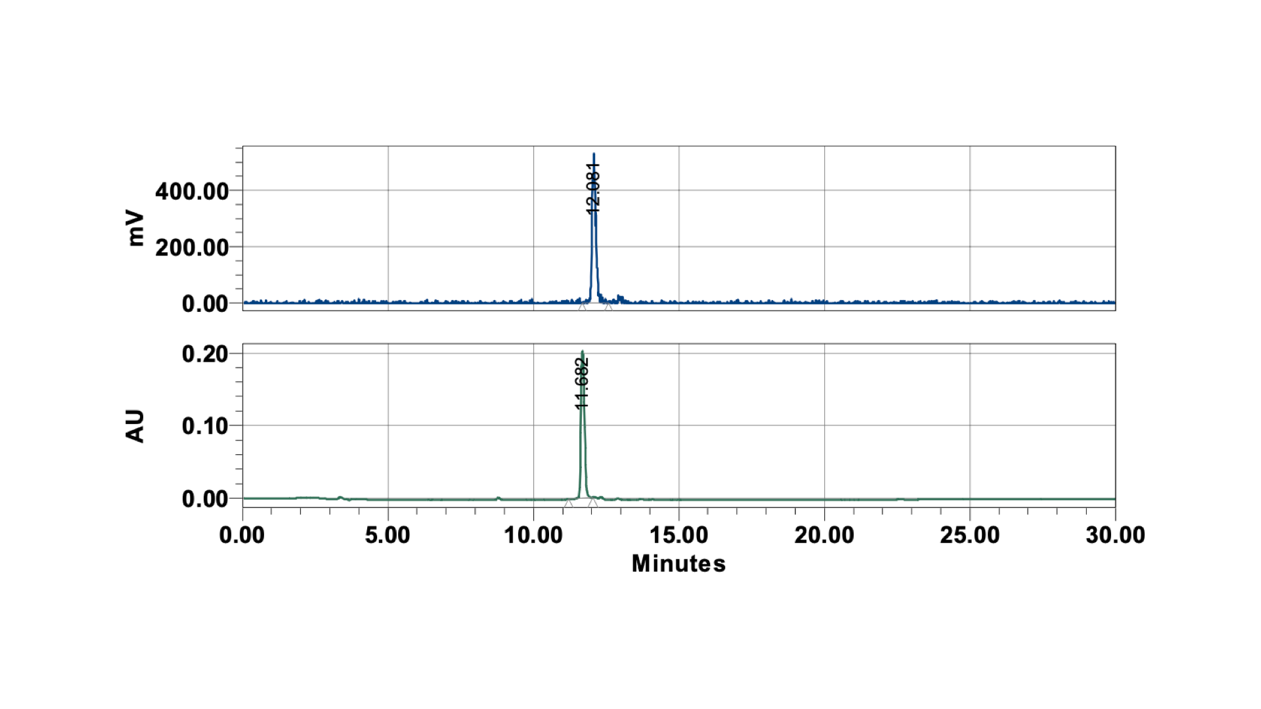


Figure S4: Radio-HPLC chromatogram (blue) and HPLC chromatogram (green) of [^177^Lu]Lu-DOTA-JR11 and ^nat^Lu-DOTA-JR11 respectively. [^177^Lu]Lu-DOTA-JR11 was ‘spiked’ with ^nat^Lu-DOTA-JR11, the co-elution of the radioactive and the non-radioactive peaks confirms the presence of identical chemical complexes.

## Stability studies in PBS and mouse serum of [^225^Ac]Ac-DOTA-JR11and [^177^Lu]Lu-DOTA-JR11


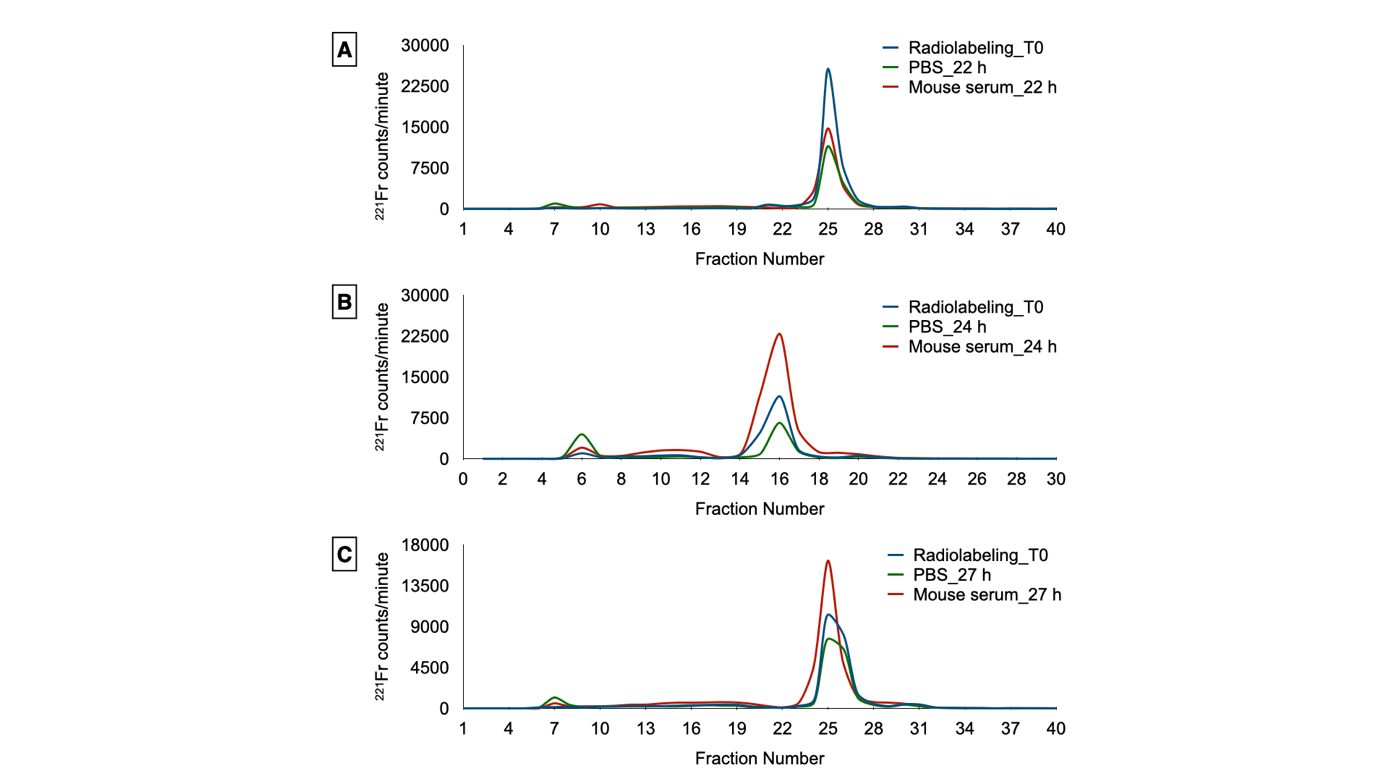


Figure S5: Radio-HPLC chromatograms of the stability studies performed for [^225^Ac]Ac-DOTA-JR11 following A) condition 1, B) condition 2 and C) condition 3 in PBS and mouse serum. The fractions of the radiolabeling and stability studies obtained for condition 2 were collected manually.


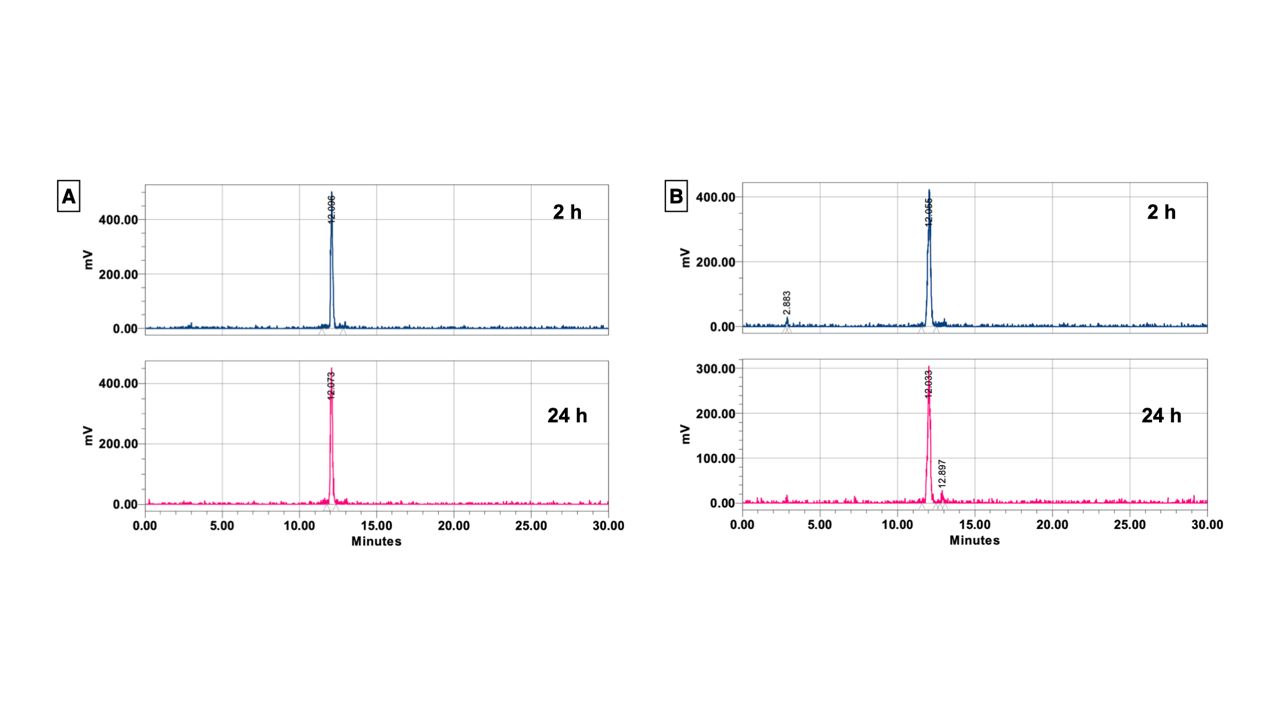


Figure S6: Radio-HPLC chromatograms of the stability studies performed for [^177^Lu]Lu-DOTA-JR11 in A) PBS and B) mouse serum at 2 and 24 h post incubation at 37 °C.

# Ex vivo studies

## Ex vivo biodistribution of [^225^Ac]Ac-DOTA-JR11

Table S1: Ex-vivo biodistribution of [^225^Ac]Ac-DOTA-JR11 (23.4 ± 1.5 kBq/0.5 nmol) at 4, 24, 48 and 72 h post-injection (n = 3 mice/group). Data are presented as the percentage of injected activity per gram of tissue (% IA/g).

| **Organ** | **4 h** | **24 h** | **24 h - Block** | **48 h** | **72 h** |
| --- | --- | --- | --- | --- | --- |
| Blood | 0.1 ± 0.0 | 0.0 ± 0.0 | 0.0 ± 0.0 | 0.0 ± 0.0 | 0.0 ± 0.0 |
| Tumor | 7.7 ± 0.9 | 6.0 ± 0.6 | 0.4 ± 0.1 | 4.5 ± 0.6 | 2.8 ± 0.5 |
| Heart | 0.0 ± 0.1 | 0.1 ± 0.0 | 0.1 ± 0.0 | 0.0 ± 0.1 | 0.0 ± 0.0 |
| Lungs | 1.4 ± 0.3 | 0.7 ± 0.2 | 0.2 ± 0.1 | 0.4 ± 0.0 | 0.3 ± 0.1 |
| Liver | 1.0 ± 0.1 | 0.8 ± 0.1 | 0.8 ± 0.0 | 1.0 ± 0.2 | 1.1 ± 0.3 |
| Spleen | 0.3 ± 0.1 | 0.3 ± 0.1 | 0.2 ± 0.1 | 0.2 ± 0.1 | 0.4 ± 0.2 |
| Stomach | 2.0 ± 0.3 | 0.8 ± 0.3 | 0.1 ± 0.0 | 0.6 ± 0.4 | 0.4 ± 0.1 |
| Intestines | 0.5 ± 0.1 | 0.2 ± 0.0 | 0.1 ± 0.0 | 0.2 ± 0.0 | 0.1 ± 0.0 |
| Pancreas | 5.1 ± 0.5 | 1.8 ± 0.3 | 0.2 ± 0.0 | 1.0 ± 0.1 | 0.8 ± 0.1 |
| Kidneys | 19.3 ± 2.6 | 14.7 ± 1.1 | 13.4 ± 1.9 | 10.6 ± 3.6 | 8.1 ± 0.3 |
| Muscle | 0.0 ± 0.0 | 0.0 ± 0.0 | 0.1 ± 0.1 | 0.0 ± 0.0 | 0.1 ± 0.1 |
| Skin | 0.5 ± 0.1 | 0.4 ± 0.1 | 0.3 ± 0.2 | 0.1 ± 0.2 | 0.3 ± 0.1 |
| Bone | 0.4 ± 0.1 | 0.3 ± 0.1 | 0.2 ± 0.1 | 0.1 ± 0.1 | 0.3 ± 0.1 |
| T/K Ratio^a^ | 0.4 ± 0.0 | 0.4 ± 0.0 |  | 0.4 ± 0.1 | 0.3 ± 0.1 |

^a^ Tumor-to-kidney ratio

Statistical analysis was performed to compare tumor, kidneys, liver and bone uptake for [^225^Ac]Ac-DOTA-JR11 and [^177^Lu]Lu-DOTA-JR11. The results revealed no significant difference of tumor uptake between both radiopeptides (Fig. S7A). Even though kidneys uptake constantly decreased overtime, the results showed that significantly higher uptake was noticed for [^225^Ac]Ac-DOTA-JR11 compared to [^177^Lu]Lu-DOTA-JR11 (19.3 ± 2.6 % IA/g and 12.6 ± 1.6 % IA/g at 4 h, 8.1 ± 0.3 % IA/g and 3.4 ± 1.4 % IA/g at 72 h, respectively) (Fig. S7B). However, a different tendency was found in the liver uptake. Although the liver uptake decreased overtime for [^177^Lu]Lu-DOTA-JR11 (0.5 ± 0.0 % IA/g at 4 h and 0.2 ± 0.0 % IA/g at 72 h p.i.), it remained steady for [^225^Ac]Ac-DOTA-JR11 (1.0 ± 0.1 % IA/g at 4 h and 1.1 ± 0.3 % IA/g at 72 h p.i.) (Fig. S7C). Significantly higher bone uptake was found for [^225^Ac]Ac-DOTA-JR11 compared to [^177^Lu]Lu-DOTA-JR11 (0.4 ± 0.1 % IA/g and 0.1 ± 0.0 % IA/g at 4 h, 0.3 ± 0.1 % IA/g and 0.0 ± 0.4 % IA/g at 24 h, respectively) (Fig. S7D).


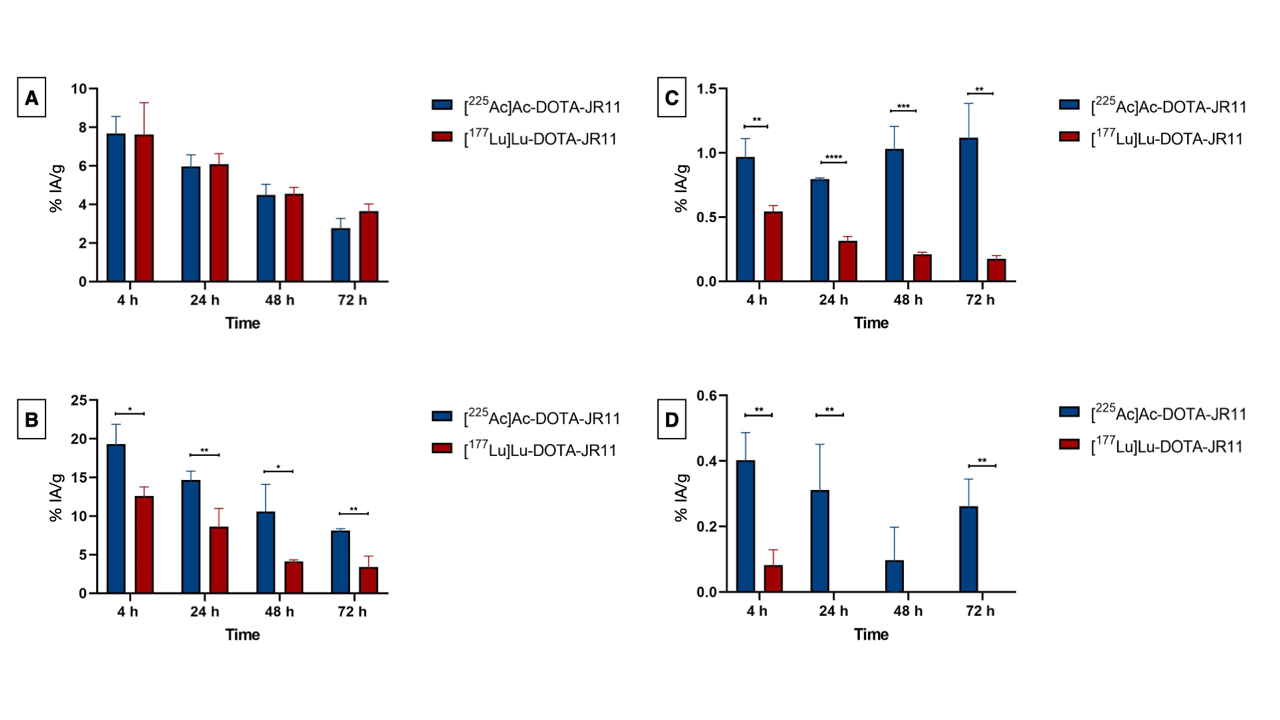


Figure S7: Comparison of A) tumor, B) kidneys, C) liver and D) bone uptake of [^225^Ac]Ac-DOTA-JR11 and [^177^Lu]Lu-DOTA-JR11 at 4, 24, 48 and 72 h p.i. Data is represented as % IA/g. * p < 0.05. ** p < 0.01. *** p < 0.0005. **** p < 0.0001. Absence of * means no significant difference found.

## Ex vivo biodistribution of [^177^Lu]Lu-DOTA-JR11

Table S2: Ex-vivo biodistribution analysis of [^177^Lu]Lu-DOTA-JR11 (5 MBq/0.5 nmol) at 4, 24, 48 and 72 h post-injection (n = 4 mice/group). Data is represented as percentage of injected activity per gram of tissue (% IA/g).

| **Organ** | **4 h** | **24 h** | **24 h - Block** | **48 h** | **72 h** |
| --- | --- | --- | --- | --- | --- |
| Blood | -0.1 ± 0.0 | -0.1 ± 0.1 | -0.1 ± 0.1 | 0.0 ± 0.0 | -0.1 ± 0.0 |
| Tumor | 8.4 ± 0.5 | 6.1 ± 0.5 | 0.4 ± 0.0 | 4.6 ± 0.3 | 3.6 ± 0.4 |
| Heart | 0.0 ± 0.0 | 0.0 ± 0.0 | 0.0 ± 0.0 | 0.0 ± 0.0 | -0.1 ± 0.0 |
| Lung | 1.3 ± 0.3 | 0.4 ± 0.0 | 0.1 ± 0.0 | 0.2 ± 0.0 | 0.1 ± 0.0 |
| Liver | 0.5 ± 0.0 | 0.3 ± 0.0 | 0.3 ± 0.0 | 0.2 ± 0.0 | 0.2 ± 0.0 |
| Spleen | 0.3 ± 0.1 | 0.1 ± 0.0 | 0.0 ± 0.0 | 0.0 ± 0.0 | 0.0 ± 0.0 |
| Stomach | 1.7 ± 0.3 | 1.5 ± 1.2 | 0.1 ± 0.1 | 0.6 ± 0.2 | 0.4 ± 0.1 |
| Intestine | 0.5 ± 0.1 | 0.2 ± 0.0 | 0.1 ± 0.0 | 0.1 ± 0.0 | 0.1 ± 0.0 |
| Pancreas | 3.5 ± 0.4 | 0.9 ± 0.3 | 0.1 ± 0.0 | 0.6 ± 0.1 | 0.3 ± 0.0 |
| Kidneys | 12.6 ± 1.2 | 8.6 ± 2.3 | 5.9 ± 1.2 | 4.1 ± 0.2 | 3.4 ± 1.4 |
| Muscle | -0.1 ± 0.0 | -0.1 ± 0.0 | -0.1 ± 0.0 | -0.1 ± 0.0 | -0.1 ± 0.0 |
| Skin | 0.3 ± 0.1 | 0.0 ± 0.1 | -0.1 ± 0.1 | -0.2 ± 0.1 | -0.4 ± 0.2 |
| Bone | 0.1 ± 0.0 | -0.6 ± 0.4 | -0.4 ± 0.5 | -0.2 ± 0.2 | -0.1 ± 0.1 |
| T/K ratio^a^ | 0.7 ± 0.0 | 0.7 ± 0.02 |  | 1.1 ± 0.1 | 1.2 ± 0.4 |

^a^ Tumor-to-kidney ratio

# Dosimetry studies

Figure S8: Time-activity curves of the organs and tissues of interest from mice injected with [^225^Ac]Ac-DOTA-JR11. Single-exponential curve fits are shown with 95% confidence intervals.

Figure S9: Time-activity curves of the organs and tissues of interest from mice injected with [^177^Lu]Lu-DOTA-JR11. Single-exponential curve fits are shown with 95% confidence intervals.
